# Validity and reproducibility of folate and vitamin B_12 _intakes estimated from a self-administered diet history questionnaire in Japanese pregnant women

**DOI:** 10.1186/1475-2891-11-15

**Published:** 2012-03-15

**Authors:** Mie Shiraishi, Megumi Haruna, Masayo Matsuzaki, Ryoko Murayama, Satoshi Sasaki, Sachiyo Murashima

**Affiliations:** 1 Department of Midwifery and Women's Health, Division of Health Sciences and Nursing, Graduate School of Medicine, The University of Tokyo, 7-3-1, Hongo, Bunkyo-ku, Tokyo 113-0033, Japan; 2 Department of Social and Preventive Epidemiology, School of Public Health, The University of Tokyo, 7-3-1, Hongo, Bunkyo-ku, Tokyo 113-0033, Japan; 3 Department of Community Health Nursing, Division of Health Sciences and Nursing, Graduate School of Medicine, The University of Tokyo, 7-3-1, Hongo, Bunkyo-ku, Tokyo 113-0033, Japan

**Keywords:** Diet history questionnaire, Pregnant women, Validation, Folate, Vitamin B_12_

## Abstract

**Background:**

No validated dietary questionnaire for assessing folate and vitamin B_12 _intakes during pregnancy is available in Japan. We evaluated the validity and reproducibility of intakes of folate and vitamin B_12 _estimated from a self-administered diet history questionnaire (DHQ) in Japanese pregnant women.

**Methods:**

A sample of 167 healthy subjects with singleton pregnancies in the second trimester was recruited at a private obstetric hospital in metropolitan Tokyo from June to October 2008 (n = 76), and at a university hospital in Tokyo from June 2010 to June 2011 (n = 91). The dietary intakes of folate and vitamin B_12 _were assessed using the DHQ. The serum concentrations of folate and vitamin B_12 _were measured as reference values in the validation study. To assess the reproducibility of the results, 58 pregnant women completed the DHQ twice within 4-5 week interval.

**Results:**

Significantly positive correlations were found between energy-adjusted intakes and serum concentrations of folate and vitamin B_12 _(r = 0.286, *p *< 0.001 and r = 0.222, *p *= 0.004, respectively). After excluding the participants with nausea (n = 121), the correlation coefficient for vitamin B_12 _increased to 0.313 (*p *= 0.001). When participants were classified into quintiles based on intakes and serum concentrations of folate and vitamin B_12 _, approximately 60% were classified in the same or adjacent quintile. The intraclass correlation coefficients of the two-time DHQ were 0.725 for folate and 0.512 for vitamin B_12 _.

**Conclusion:**

The present study indicated that the DHQ had acceptable validity and reproducibility for assessing folate and vitamin B_12 _intakes in Japanese pregnant women.

## Background

Low birth weight is a major cause of perinatal morbidity and low birth weight babies are susceptible to cardiovascular disease in later life [1]. The incidence of low birth weight increased in Japan from 6.3% in 1990 to 9.6% in 2007 [2]. One possible reason for the low birth weight is low intake of micronutrients during pregnancy [3,4]. For instance, folate and vitamin B_12 _deficiency can lead to retardation of fetal development through hyperhomocysteinemia, which negatively affects DNA methylation and cell proliferation [5-8]. Assessment of an individual's nutritional status including folate and vitamin B_12 _is therefore important to prevent the harmful effects of improper nutrient intake on pregnancy outcome.

The methods used for assessment of dietary intake often include weighed food records, measurement of certain blood markers, and 24-hour urine analysis [9]. However, these methods may be costly and can impose a significant burden on participants. Therefore, easier and less onerous methods are needed for screening and large-scale research studies, such as the dietary assessment questionnaire. This convenient method is used commonly in epidemiological studies to identify individuals with low and high nutrient intake [9]. However, no validated dietary assessment questionnaire exists in Japan for pregnant women, making it difficult to implement large interventional studies and epidemiologic studies regarding prenatal nutrition.

A self-administered diet history questionnaire (DHQ) developed in Japan to evaluate nutrient intakes in both healthy individuals and high-risk populations has been validated only in non-pregnant populations [10,11]. A separate validation study of the DHQ for pregnant women is therefore required, particularly given that dietary habits are likely to change in both pregnant women and their babies, and dietary intake is affected by physical conditions associated with pregnancy, and changes in appetite and feeling [12].

The present study was designed to assess the relative validity and reproducibility of the DHQ among Japanese pregnant women, using serum biomarkers of folate and vitamin B_12_. The study also compared folate and vitamin B_12 _intakes estimated from two-time DHQ.

## Methods

### Overview of recruitment and study design

The present study was conducted at a private obstetric hospital (S hospital) in metropolitan Tokyo, Japan, between June and October 2008, and at a university hospital (T hospital) in Tokyo between June 2010 and June 2011. Neither hospital routinely conducts individualized guidance of nutrient intake for low-risk pregnant women. For this study, healthy Japanese women with singleton pregnancies were surveyed during the second trimester. The exclusion criteria included patients with hyperemesis and hypertension to avoid their burden of participation in the study in consideration of their symptoms. We also excluded women with diabetes and those enrolled in formal nutrition training because they had done other interviews regarding their intakes and had received nutritional intervention. Other exclusion criteria included age younger than 20 years, poor ability reading Japanese, and intake of supplements including folic acid and vitamin B_12_. All women underwent ultrasonography at 8-12 weeks gestation to allow accurate dating of gestation. All participants were provided with detailed information about the study protocol, and each gave a written informed consent. The Ethics Committee of the Graduate School of Medicine at the University of Tokyo approved the study procedures and protocol.

Each participant completed the questionnaire while waiting for a pregnancy checkup (S hospital: 24-27 weeks gestation, T hospital: 19-23 weeks gestation). Participants who did not have sufficient time to complete the questionnaires filled them out after returning home, and submitted them by mail. We resolved missing and unclear data directly when questionnaires were submitted to researchers or by telephone interview, and reviewed participants' medical charts to obtain information on the pregnancy. To reduce subjects' burden, we drew non-fasting blood samples at the hospitals during collection of biological samples for routine blood tests that were conducted in association with a normal pregnancy health examination (S hospital: 24-27 weeks gestation; T hospital: 19-23 weeks gestation). The participants answered questionnaires including the DHQ on the day of blood sample collection or within 7 days after the blood sample collection.

To assess the reproducibility of the DHQ, pregnant women at 15-19 weeks gestation were recruited at T hospital between February and April, 2011 and asked to complete the DHQ twice in the second trimester; the first DHQ was completed at recruitment and the second DHQ 4-5 weeks later.

### Diet history questionnaire

The DHQ was designed to assess dietary intake over the previous month using a questionnaire developed and validated for the Japanese adult population [10,11]. The DHQ is a 22-page structured questionnaire that measures the daily intake of 150 foods, and calculates the intakes of energy, folate, and vitamin B_12 _using an ad hoc computer algorithm based on the Japanese standard food composition tables [13]. Items and portion sizes were derived from primary data of the National Nutrition Survey of Japan and from various Japanese recipe books for Japanese dishes [10]. The daily intakes of vitamins were calculated as nutritional amounts in the foods consumed multiplied by the frequency and portion size. Eight frequency responses were listed ranging from more than twice per day to almost never. Five portion size responses were listed from less than half to more than 1.5 times a general portion size. These standard values were established as part of the initial development of the DHQ for non-pregnant populations.

We excluded participants who reported an extremely unrealistic energy intake; that is, the reported energy intake was less than half the energy requirement for the lowest physical activity category or more than 1.5 times the energy requirement for moderate physical activity according to the "Dietary Reference Intakes for Japanese" [14,15].

### Biological markers

Blood samples were centrifuged for 10 minutes at 3000 rpm to separate serum within 6 hours after blood collection, and the serum samples were stored at -80°C until analysis. The samples were measured within 3 months after collection. Serum concentrations of folate and vitamin B_12 _were assayed by chemiluminescent immunoassay using ADVIA Centaur^® ^(Siemens AG, Munich, Germany) with Chemilumi-ACS folate II and Chemilumi-ACS vitamin B_12 _kits (Siemens Healthcare Diagnostics Co. Ltd., Tokyo, Japan). These assays were conducted by Mitsubishi Kagaku Bio-Chemical Laboratories, Tokyo, Japan.

### General questionnaires and data processing

We collected demographic and lifestyle information from the study participants, such as age, gestational age and educational levels, using a self-administered questionnaire. Education level was categorized as < 13, 13-15, or > 15 completed schooling years. Prepregnancy body mass index (BMI) was calculated from self-reported prepregnancy weight and height. The participants were classified as underweight (BMI < 18.5 kg/m^2 ^), normal weight (18.5 ≤ BMI < 25.0 kg/m^2 ^) and overweight or obese (BMI ≥ 25.0 kg/m^2s^) based on the criteria of the World Health Organization. The pregnant women were also asked about nausea and vomiting during pregnancy.

### Statistical analysis

#### Validation study

We assumed that the level of validity between intakes estimated from the DHQ and their corresponding serum concentrations was more than r = 0.40, with 80% power and a 5% significance level. A sample size of more than 123 would be required to detect a minimally acceptable level of r = 0.25 [16]. We compared parameters between the two hospitals using Student's t-test or the chi-square test. The Shapiro-Wilk test was used to test for normal distribution of the continuous variables. We used the density method for energy-adjustment of vitamins intake. Partial correlation analyses were conducted to test for associations between dietary intakes and serum concentrations of folate and vitamin B_12_, adjusting for research settings. These analyses were conducted after all variables were log-transformed to achieve normal distributions.

All pregnant women were classified into quintiles according to their intakes and serum concentrations for each vitamin. Agreement and discordance in quintile ranking was assessed as the percentage classification for the pregnant women based on dietary intake and corresponding serum concentration in the same, adjacent, and opposite quintiles. We also calculated mean vitamins intakes estimated from the DHQ in each quintile of serum concentrations. Linear regression analyses were performed to test for significant linear trends between intake and serum concentration, adjusting for research settings.

#### Reproducibility study

We assumed that the level of reliability between the DHQ at two points was more than r = 0.60, with 80% power and a 5% significance level. A sample size of more than 47 would be required to detect a minimally acceptable level of reliability of r = 0.40 [16].

The Shapiro-Wilk test was used to test for normal distribution of the continuous variables. The mean intakes estimated from the two-time DHQ were compared using the paired t-test, after all variables except energy intake, were log-transformed to achieve normal distribution. In addition, we calculated the Intraclass Correlation Coefficients of intake estimated from the two-time DHQ. All pregnant women were also classified into quintiles according to folate and vitamin B_12 _intakes estimated from the two-time DHQ. Agreement and discordance in quintile ranking was assessed as the percentage classification for pregnant women based on the two-time DHQ in the same, adjacent, and opposite quintiles. We also calculated mean vitamins intakes estimated from the second DHQ in each quintile based on the first DHQ. Linear regression analyses were performed to test for significant linear trends between estimated intakes from the two-time DHQ.

Bland-Altman plots were used to illustrate the difference between the two-time DHQ against the mean intakes by the two-time DHQ [17]. The upper and lower lines represented the upper and lower 95% limits of agreement (mean difference ± 1.96 SD).

All statistical analyses were conducted using The Statistical Package for Social Sciences for Windows, version 15.0 (SPSS Japan Inc.). All statistical tests were two-sided and *P *values less than 0.05 were considered statistically significant.

## Results

### Validation study

We recruited 358 pregnant women (S hospital: 128, T hospital: 230) and 322 (89.9%; S hospital: 114, T hospital: 208) of these gave their written informed consent. We excluded 128 pregnant women who were taking folic acid or vitamin B_12 _supplements (S hospital: 27, T hospital: 101) and further 27 women for the following reasons: 12 had missing data; 8 had a severe under-reported energy intake; and 7 had outlier data such as high serum concentrations despite low intakes. Our final analysis was therefore of data from 167 healthy singleton pregnant women (46.6%; S hospital: 76, T hospital: 91).

Table 1 summarizes the characteristics of the participants. Participants in the T hospital had a higher mean age than those in the S hospital, and there were significant differences in education level and household income between the two hospitals.

**Table 1:** Characteristics of participants

	Validation study							Reproducibility study (n = 58)	
			
	All participants (n = 167)		S Hospital (n = 76)		T Hospital (n = 91)				
			
	Mean ± SD or n (%)		Mean ± SD or n (%)		Mean ± SD or n (%)		*p*^1^	Mean ± SD or n (%)	
Age [years]	32.4 ± 4.9		30.5 ± 5.0		34.1 ± 4.1		< 0.001	33.8 ± 4.2	
Parity: Primigravida [n (%)]	80	(48)	34	(45)	46	(51)	ns	38	(66)
Currently married [n (%)]	166	(99)	75	(99)	91	(100)	ns	58	(100)
Education [n (%)]									
< 13 years	32	(19)	25	(33)	7	(8)	< 0.001	3	(5)
13-15 years	75	(45)	37	(49)	38	(42)		24	(41)
> 15 years	60	(36)	14	(18)	46	(51)		31	(53)
Household income (Japanese yen/year) [n (%)]									
< 5,000,000	59	(35)	38	(50)	21	(23)	< 0.001	7	(12)
5,000,000-9,000,000	70	(42)	32	(42)	38	(42)		27	(47)
> 9,000,000	38	(23)	6	(8)	32	(35)		24	(41)
Height [cm]	158.7 ± 5.7		157.8 ± 5.2		159.5 ± 5.9		0.048	159.1 ± 5.4	
Prepregnancy BMI^2 ^[kg/m^2^]	20.5 ± 2.3		20.6 ± 2.3		20.3 ± 2.4		ns	20.2 ± 3.0	
BMI < 18.5 [n (%)]	28	(17)	8	(11)	20	(22)	ns	17	(29)
18.5 ≤ BMI < 25.0 [n (%)]	131	(78)	65	(86)	66	(73)		38	(65)
BMI ≥ 25.0 [n (%)]	8	(5)	3	(4)	5	(6)		3	(5)
Working [n (%)]	66	(40)	21	(28)	45	(50)	0.004	22	(38)
Regular smoker during pregnancy [n (%)]	3	(2)	1	(1)	2	(2)	ns	1	(2)
Regular alcohol drinker during pregnancy [n (%)]	5	(3)	2	(3)	3	(3)	ns	2	(3)
Nausea during the study period [n (%)]	46	(28)	15	(20)	31	(34)	0.039	10	(17)
Gestational age [weeks]	22.6 ± 2.8		25.4 ± 1.0		20.3 ± 1.2		< 0.001		
Gestational age at first survey [weeks]								16.9 ± 1.5	
Gestational age at second survey [weeks]								21.0 ± 1.3	

^1^Student's *t*-test or the chi-square test ^2^BMI: body mass index

Table 2 summarizes the mean vitamins intake estimated from the DHQ and mean serum concentrations. Significant differences were found in daily folate intakes, energy-adjusted folate intakes, serum folate concentrations, and serum vitamin B_12 _concentrations between the two hospitals.

**Table 2:** Mean intakes estimated from a self-administered diet-history questionnaire and mean serum concentrations

	All participants (n = 167)	S Hospital (n = 76)	T Hospital (n = 91)	
		
	Mean ± SD	Mean ± SD	Mean ± SD	*p*^1^
**Intakes**				
Energy [kcal/day]	1823 ± 373	1832 ± 377	1815 ± 372	0.770
Folate [μg/day]^2^	261 ± 107	215 ± 76	299 ± 114	< 0.001
[μg/1,000 kcal]^2^	142 ± 48	117 ± 30	164 ± 50	< 0.001
Vitamin B_12 _[μg/day]^2^	5.74 ± 3.10	5.66 ± 3.54	5.80 ± 2.70	0.328
[μg/1,000 kcal]^2^	3.10 ± 1.35	3.01 ± 1.49	3.17 ± 1.22	0.200
**Serum concentrations**				
Folate [ng/ml]^2^	6.57 ± 2.80	5.25 ± 2.52	7.67 ± 2.55	< 0.001
Vitamin B_12 _[pg/ml]^2^	268 ± 99	199 ± 69	325 ± 83	< 0.001

^1^Student's *t*-test

^2^The analyses were conducted after log-transformation of the variables

Both the daily intakes and energy-adjusted intakes of the two vitamins correlated positively with the corresponding serum concentrations (Table 3). When participants with reported nausea were excluded (n = 121), the correlation coefficient between energy-adjusted intake and serum concentration of vitamin B_12 _increased from 0.222 to 0.313, whereas the correlation coefficient for folate decreased slightly. The mean dietary intakes in quintiles based on serum concentrations are presented in Table 4. When participants were classified into quintiles based on intakes and serum concentrations of folate and vitamin B_12_, approximately 60% fell into the same or adjacent quintiles. Meanwhile, 4-5% of the participants were classified in the opposite quintile.

**Table 3:** Correlation coefficients between intakes and serum concentrations of folate and vitamin B_12_

	Corresponding serum concentrations			
	
	All participants (n = 167)		Participants without nausea (n = 121)^2^	
	
	r	*p*^1^	r	*p*^1^
**Intakes**^3^				
Folate [μg/day]	0.251	0.001	0.245	0.007
[μg/1,000 kcal]	0.286	< 0.001	0.280	0.002
Vitamin B_12 _[μg/day]	0.174	0.025	0.249	0.006
[μg/1,000 kcal]	0.222	0.004	0.313	0.001

^1^Partial correlation analysis. Adjusted for research settings

All variables were log-transformed before analyses

^2^The participants with nausea were excluded from the analyses

^3^Folate and vitamin B_12 _intakes were assessed by a self-administered diet history questionnaire (DHQ)

**Table 4:** Mean intakes in each quintile based on corresponding serum concentrations and cross-classification between quintiles of intakes and serum concentrations (n = 167)

	Corresponding serum concentrations					Agreement (% classified into the same or adjacent quintile)^1^	Discordance (% classified into the opposite quintile)^1^	Linear trend^2^ *p*
				
	1^st ^quintile	2^nd ^quintile	3^rd ^quintile	4^th ^quintile	5^th ^quintile			
				
	Mean ± SD	Mean ± SD	Mean ± SD	Mean ± SD	Mean ± SD			
**Intakes**								
Folate [μg/day]	209 ± 96	258 ± 91	249 ± 91	286 ± 101	296 ± 134	64.7	4.8	0.003
[μg/1,000 kcal]	115 ± 39	145 ± 46	139 ± 49	145 ± 35	164 ± 58	61.1	3.6	0.002
Vitamin B_12 _[μg/day]	4.62 ± 2.43	6.36 ± 4.20	6.02 ± 2.91	4.88 ± 1.81	6.82 ± 3.17	54.5	4.2	0.029
[μg/1,000 kcal]	2.57 ± 1.29	3.23 ± 1.68	3.20 ± 1.24	2.90 ± 0.84	3.58 ± 1.41	59.9	4.2	0.005

Folate and vitamin B_12 _intakes were assessed by a self-administered diet history questionnaire (DHQ)

^1^Agreement and discordance in quintile ranking was assessed as the percentage classification for the pregnant women based on intake and corresponding serum concentration in the same, adjacent, and opposite quintiles.

^2^Linear regression analyses were performed to test for significant linear trends between intakes and serum concentrations, adjusting for research settings. The dependent variables were log-transformed

### Reproducibility study

A total of 64 pregnant women in the T hospital completed two DHQ rounds. Of these, we excluded 6 pregnant women from the analysis: 3 dropped out, 1 had missing data, and 2 had a severe under-reported energy intake. Ultimately, data of 58 women were analyzed to assess the DHQ reproducibility. Of the 58 participants, 13 were included in the validation study. There were no significant differences in the characteristics between participants in the reproducibility study and T hospital participants in the validation study (Table 1).

No significant differences were found in the mean intakes of folate and vitamin B_12 _estimated from the two-time DHQ (Table 5). Intraclass correlation coefficients of daily intakes between two-time DHQ were 0.725 for folate and 0.512 for vitamin B_12_. When each intake estimated from the two-time DHQ was classified into quintiles, more than 70% of the pregnant women were classified in the same or adjacent quintiles. The percentage of pregnant women classified into the opposite quintile was 0% for folate and 1.7% for vitamin B_12 _(Table 6).

**Table 5:** Mean daily intakes and Intraclass Correlation Coefficients between intakes estimated from the two-time DHQ (n = 58)

	1st DHQ	2nd DHQ		1st DHQ + 2nd DHQ Intraclass Correlation Coefficient
			
	Mean ± SD	Mean ± SD	*p*^1^	
Energy [kcal/day]	1727 ± 407	1771 ± 399	0.523	0.599
Folate [μg/day]^2^	292 ± 120	279 ± 112	0.257	0.725
Vitamin B_12 _[μg/day]^2^	5.37 ± 2.79	5.91 ± 2.95	0.052	0.512

DHQ: self-administered diet history questionnaire

^1^The paired *t*-test

^2^The analyses were performed after log-transformation of the variables to achieve normal distribution

**Table 6:** Mean intakes estimated from the 2nd DHQ across quintiles of intakes estimated from the 1st DHQ (n = 58)

	1st DHQ					Agreement (% classified into the same or adjacent quintile)^1^	Discordance (% classified into the opposite quintile)^1^	Linear trend^2^ *p*
				
	1^st ^quintile	2^nd ^quintile	3^rd ^quintile	4^th ^quintile	5^th ^quintile			
				
	Mean ± SD	Mean ± SD	Mean ± SD	Mean ± SD	Mean ± SD			
**2nd DHQ**								
Energy [kcal/day]	1452 ± 498	1786 ± 292	1591 ± 227	1908 ± 300	2120 ± 323	70.7	1.7	< 0.001
Folate [μg/day]^3^	185 ± 44	241 ± 37	243 ± 60	288 ± 63	447 ± 126	81.1	0	< 0.001
Vitamin B_12 _[μg/day]^3^	4.44 ± 2.32	5.03 ± 2.41	5.27 ± 1.51	6.16 ± 1.78	8.77 ± 4.36	77.6	1.7	< 0.001

DHQ: self-administered diet history questionnaire

^1^Agreement and discordance in quintile ranking was assessed as the percentage classification for the pregnant women based on the two-time DHQ in the same, adjacent, and opposite quintiles

^2^Linear regression analyses were performed to test for significant linear trends between estimated intakes from the two-time DHQ

^3^Linear regression analyses were conducted after log-transformation of the variables

**Figure 1:**
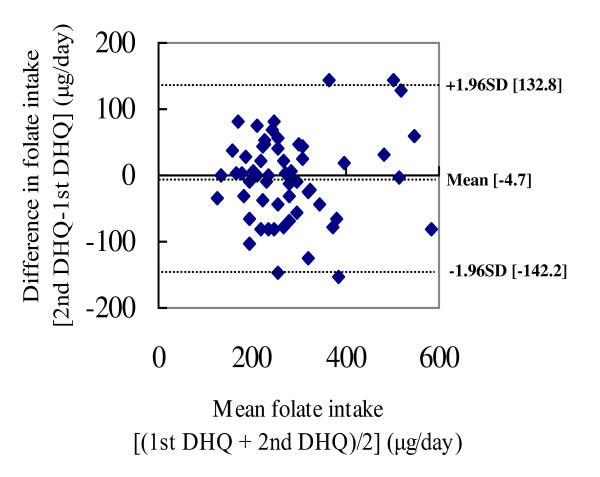
**Bland-Altman plot comparing folate intake estimated from the two-time DHQ**. DHQ: self-administered diet history questionnaire. The difference between folate intakes estimated from the two-time DHQ for each person (*y*-axis) is plotted against the mean folate intake averaged from the two-time DHQ (*x*-axis). The mean difference and the upper and lower 95% limits of agreement are shown by dotted lines.

**Figure 2:**
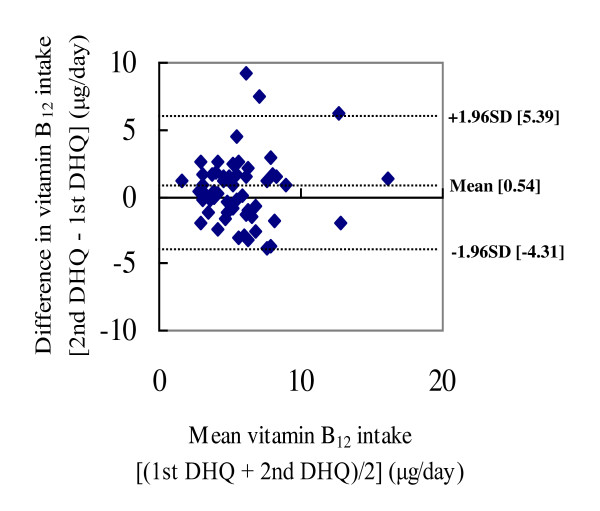
**Bland-Altman plot comparing vitamin B_12 _intake estimated from the two-time DHQ**. DHQ: self-administered diet history questionnaire. Difference between vitamin B_12 _intakes estimated from the two-time DHQ for each person (*y*-axis) is plotted against the mean vitamin B_12 _intake averaged from the two-time DHQ (*x*-axis). The mean difference and the upper and lower 95% limits of agreement are shown by dotted lines.

The Bland-Altman plots of folate and vitamin B_12 _showed that most participants were in the accepted range of agreement, but a few women showed differences between the intakes estimated from the two-time DHQ beyond the accepted ranges (Figure 1 and 2).

## Discussion

To our knowledge, this is the first study to validate the dietary intake of folate and vitamin B_12 _in Japanese pregnant women using quantitative biological markers. Our findings showed correlation coefficients of 0.222-0.313 between energy-adjusted intakes and serum concentrations of folate and vitamin B_12_. In previous studies of non-pregnant women, the reported correlation coefficients ranged from 0.20 to 0.51 for folate and from 0.20 to 0.27 for vitamin B_12_[18-21]. Meanwhile, a Norwegian study of pregnant women found a correlation coefficient of 0.26 for folate between intakes and serum concentrations [22]. In general, correlation coefficients of more than 0.50 are considered as closely correlated, 0.30-0.50 as acceptable, and less than 0.30 as poorly correlated [23]. However, the correlations between intakes and biomarkers are often lower during pregnancy than in the non-pregnant period. This is because some nutrients are required for continuation of pregnancy and fetal development, and a greater intraindividual variability in intakes can occur during pregnancy than non-pregnancy periods [12,24,25]. Compared with the criteria of Ortiz-Andrellucchi et al. and another study on pregnancy [22,23], the present study showed acceptable validity for assessing energy-adjusted intakes of folate and vitamin B_12_. Using the energy-adjusted values is recommended in epidemiological studies because energy adjustment can reduce intraindividual measurement errors [26]. Therefore, the DHQ can be used in epidemiological studies for Japanese pregnant women.

The correlation coefficient between the intake and serum concentration of vitamin B_12 _increased in participants without nausea in the present study. Nausea and vomiting in pregnancy often alter food consumption, and a previous study showed that pregnant women with nausea consumed less meat products, which contain large amount of vitamin B_12_, than those without nausea, whereas consumption of vegetables and fruits, which contain large amount of folate, were not affected by nausea [27]. Since the degree of nausea varies daily and as gestation progresses, food selections would change over time. Therefore, among pregnant women with nausea, assessing habitual consumption of foods that can be affected by nausea might be difficult. In the present study, the correlation coefficient and the cross-classification analysis showed that the DHQ provided a reasonable validity for assessing energy-adjusted intakes of vitamin B_12_, regardless of the possibility of nausea. However, habitual vitamin B_12 _intakes estimated from the DHQ in pregnant women with nausea need to be interpreted carefully because the correlation coefficient of vitamin B_12 _in all participants including pregnant women with nausea was lower than the acceptable level of 0.30 [23].

In the present study, we excluded pregnant women taking supplements including folic acid and vitamin B_12_, which was 49% of the recruited pregnant women in the T hospital. This percentage in the second trimester was twice as high as that in the S hospital and in other studies of Japanese pregnant women [28,29]. The attitudes toward dietary intakes and supplementation might have been affected by the difference between hospitals. However, serum concentrations of folate and vitamin B_12 _in the present study were similar to other studies among non-supplement users [30,31]. Therefore, we considered that our participants were representative of Japanese pregnant women in the general population in terms of folate and vitamin B_12 _status.

Good correlation coefficients achieved in the reproducibility study of the dietary assessment questionnaire range from 0.50 to 0.70 [32]. Since the correlation coefficient for folate and vitamin B_12 _was 0.725 and 0.512, respectively, in the present study, the intakes estimated from the two-time DHQ showed good reproducibility among pregnant women. Meanwhile, mean vitamin B_12 _intake from the first DHQ was lower than that from the second DHQ, although non-significantly. In addition, the Bland-Altman plots showed an unacceptable difference between the two-time DHQ in a few pregnant women. We speculate that this result for vitamin B_12 _might be partly due to the altered food selection associated with nausea and vomiting, as discussed for the validation study [27]. Even if participants had no nausea or vomiting when completing the DHQ, some might have had such symptoms in the object period of the first DHQ because the period included 11-12 gestation weeks when most pregnant women suffer nausea and vomiting [33]. On the other hand, cross-classification analyses and linear trends indicated that the DHQ could determine pregnant women with low and high intakes of both folate and vitamin B_12_. The fact that the reproducibility of the DHQ during pregnancy was established even in such a slightly unstable period indicated that the DHQ is probably a reliable dietary assessment tool throughout pregnancy.

The current study had several limitations. First, the characteristics of the participants were likely to be biased because one of the research hospitals was a university hospital in an urban area. Second, prepregnancy weight and height were self-reported. Therefore, the reporting bias might affect the values. Third, we did not test for 5,10-methylenetetrahydofolate reductase (MTHFR) genotypes, which affects folate metabolism. Thirteen to sixteen percent of Japanese people have the TT genotype, with lower circulating folate levels [34,35]. Therefore, the relationship between intake and serum concentration of folate might vary depending on MTHFR genotype. Fourth, we could not obtain detailed information regarding intakes of fortified foods with folic acid. In Japan, a few food products such as sweets, eggs, milk, and candy are fortified with folic acid. Accordingly, this might have affected the result of the present study. Finally, full diet composition might not be estimated from the DHQ due to variations in capacity of the described food and seasonal change.

## Conclusion

The present study confirmed that the DHQ had acceptable validity and reproducibility for assessing folate and vitamin B_12 _intakes in Japanese pregnant women. This result would be useful for nutritional intervention studies and epidemiological studies of the relationship between pregnancy complications and intake of folate and vitamin B_12 _in Japan.

## Abbreviations

DHQ: Self-administered diet history questionnaire; BMI: Body mass index; MTHFR: Methylenetetrahydrofolate reductase.

## Competing interests

The authors declare that they have no competing interests.

## Authors' contributions

All authors conceived and designed the study. MS performed the statistical analyses. MS, MH, MM and SS interpreted the results. MS drafted the manuscript. All authors revised the manuscript for intellectual content, and read and approved the final manuscript.
